# A questionnaire survey on the implementation of palliative care in the emergency department in China

**DOI:** 10.1186/s12904-024-01407-5

**Published:** 2024-03-09

**Authors:** Yan Li, Huadong Zhu, Jun Xu, Jing Yang

**Affiliations:** Department of Emergency Medicine, State Key Laboratory of Complex Severe and Rare Diseases, Peking Union Medical College Hospital, Chinese Academy of Medical Science and Peking Union Medical College, No.1 Shuaifuyuan Wangfujing, Dongcheng District, Beijing, China

**Keywords:** Poor knowledge, Palliative care, End-stage disease, Resuscitation room, Emergency department

## Abstract

**Objectives:**

This study was conducted to characterize the need for palliative care and its effect on patients with end-stage disease in the emergency department (ED).

**Design:**

This was a prospective cohort study. A questionnaire survey was administered to patients with end-stage disease who were admitted to the resuscitation room of the ED and expected to live less than 6 months.

**Results:**

A total of 82 of 2095 patients admitted to the resuscitation room were included. Only 1 (1.22%) patient had ever received palliative care before admission. Nine patients received palliative care consultation after admission, and they were more likely to select medical places of death accompanied by their families and do not resuscitate orders at the end of life after consultation (*P* < 0.05). Whether the disease had previously been actively treated and the number of children impacted the choice of treatment at the end of life (*P* < 0.05).

**Conclusions:**

Among patients with end-stage disease admitted to the ED, knowledge of palliative care was lacking. Palliative care could help them avoid the damage caused by pointless resuscitation.

**Supplementary Information:**

The online version contains supplementary material available at 10.1186/s12904-024-01407-5.

## Introduction

In China, the resuscitation room is used for cardiopulmonary resuscitation, endotracheal intubation, and advanced cardiac support. For example, patients who may require tracheal intubation, such as those with disorders of consciousness and inability to manage their airways, need to be admitted to the resuscitation room [1]. Most of the time, patients are admitted to the resuscitation room of the emergency department (ED) for active and life-saving treatment. However, for those with end-stage disease, no effective medical methods are available to save their lives [2]. Palliative care, which is intended to improve the quality of life of patients and their families’ facing problems associated with life-threatening illness, could be more effective than treatment in the resuscitation room [3]. Most Eds cannot provide a quiet and comfortable environment for patients.

Palliative care has been one of the most rapidly growing fields of health care in the past decade [4]. However, the knowledge of emergency patients and their families about palliative care in China is unclear. A previous study showed that patients with advanced cancer continued to present to the ED despite recommendations for early delivery of palliative care [5, 6]. In traditional Chinese culture, mentioning death can be considered taboo or even disrespectful, leading to a tendency to avoid conversations related to end-of-life decisions including palliative care [7]. In this study, we surveyed patients with end-stage disease and their families about their knowledge of palliative care and changes in choice before and after being surveyed.

## Materials and methods

### Study design and data sources

Patients who were admitted to the resuscitation room of the ED at Peking Union Medical College Hospital (PUMCH) from December 1, 2021, to June 1, 2022, and their families were enrolled in this study. As a referral center offering diagnostic and therapeutic care for complex and rare disorders in China, PUMCH is a tertiary care hospital in Beijing, and the patients come from all over the country. The resuscitation room of the ED has 21 beds that are closed off to visitors. The inclusion criteria for patients were as follows: suffering from an uncurable end-stage disease and a life expectancy of less than 6 months (determined by attending doctors from at least two or more departments) [8]; agreed participating in the study.

A separate online data collection questionnaire was used for the survey through a web platform (www.wjx.cn) that permits centralized data collection and limits duplicate mobile numbers (the English version of the questionnaire can be found in the Supplementary Material). The survey was a standard questionnaire that was constructed from themes based on a review of the literature and the study aim. The survey included two parts. Part 1 had 33 questions and consisted of four categories: category 1 included 5 questions that addressed demographic data (patient ID, sex, age, education level and religion); category 2 included 4 questions referring to the end-stage disease of the patient (type, treatment and end-stage care); category 3 included 7 questions that concentrated on the patient’s lifestyle and knowledge about the disease; and category 4 included 8 questions that concentrated on the patient’s family and financial support. The last category of seven questions referred to who made the decision and whether they needed the help of the hospice team. Part 2 evaluated health-related quality of life (HRQOL). HRQOL was measured using the Chinese version of the Medical Outcome Study 36-item Short Form Health Survey (MOS SF-36) [9, 10]. The MOS SF-36 consists of 36 items covering 8 areas, namely, physical function, physical roles, physical pain, overall health status, vitality, social function, emotional roles, and mental health.

This study was conducted among the patients and their families if the patients were awake and among the families only if the patients were in a coma. This study was approved by the Ethics Committee on Human Experimentation of Peking Union Medical College Hospital (reference number: JS-2898).

### Statistics

All the statistical analyses were completed with Statistical Package for the Social Sciences (IBM SPSS Statistics for Windows, IBM Corp., Version 21.0, Armonk, NY). The normality of the data distribution was assessed using the Kolmogorov‒Smirnov test. A group t test was applied for normally distributed data, and the Mann‒Whitney U test was applied for nonnormally distributed data. The chi-square test was used for comparisons of two or more rates or components. Analyses are presented as two-sided comparisons. A P value less than 0.05 was considered to indicate statistical significance.

## Results

### 1. General patient information

In total, 2095 patients were admitted to the resuscitation room of the ED during the study period. Eighty-two patients met the inclusion criteria and were enrolled in this study. The general information of the patients, including age, sex, education level, religious beliefs, family background, type of end-stage disease and perceptions of the disease status of the patients and families, is reported in Table 1.

**Table 1:** The basic information of the patients

	Total (*n* = 82)
**Age**	64.30 ± 14.38
**Sex (Male/Female)**	46/36
**Education level of the patients**	
High school degree or below, n (%)	54 (65.85%)
College degree or above, n (%)	28 (34.15%)
**Religious beliefs of the patients**	
Member of Communist Party of China, n (%)	20 (24.39%)
Buddhism, n (%)	2 (2.44%)
Christian, n (%)	3 (3.66%)
None, n (%)	57 (69.51%)
**Type of end-stage disease**	
Malignant tumor, n (%)	64 (78.05%)
Organ failure, n (%)	9 (10.98%)
Advanced age, n (%)	11 (13.41%)
No ability to take care of themselves, n (%)	15 (18.29%)
Others (%)	3 (3.66%)
**The major end-stage disease had previously been actively treated**	
Yes, n (%)	64 (78.05%)
No, n (%)	18 (21.95%)
**Consciousness of the patient**	
Aware, n (%)	49 (59.76%)
Somnolence, n (%)	5 (6.10%)
Confusion, n (%)	10 (12.20%)
Coma, n (%)	18 (21.95%)
**Whether the patient was aware of his or her disease**	
Absolutely yes, n (%)	45 (54.88%)
Partially yes, n (%)	13 (15.85%)
Absolutely no, n (%)	7 (8.54%)
Unknowing, n (%)	17 (20.73%)
**Whether a will had been made**	
Yes, n (%)	15 (18.29%)
No, n (%)	67 (81.71%)
**Whether the patient had a strong desire for treatment**	
Yes, n (%)	42 (51.22%)
No, n (%)	40 (48.78%)
**Marital status**	
Married	57 (69.51%)
Wid	18 (21.95%)
Unmarried	4 (4.88%)
Divorced	3 (3.66%)
**Number of children**	
0	7 (8.54%)
1	43 (52.44%)
2	20 (24.39%)
More than 2	12 (14.63%)
**Age of the children**	
≥ 18 years	65 (79.27%)
< 18 years	17 (20.73%)
**Live alone**	
Yes, n (%)	3 (3.66%)
No, n (%)	79 (96.34%)
**MOS SF-36 score**	
< 50, n (%)	66 (80.49%)
≥ 50, n (%)	16 (19.51%)
**Time of diagnosing terminal disease**	
< 1 month, n (%)	10 (12.20%)
1 month to 1 year, n (%)	28 (34.15%)
> 1 year, n (%)	44 (53.66%)
**Expected life**	
< 1 month, n (%)	58 (70.73%)
1–6 months, n (%)	24 (29.27%)
**Whether the family members were clear about the patients’ expected life before admission**	
Yes, n (%)	43 (52.44%)
No, n (%)	39 (47.56%)

A normal score lower than 50 indicated poorer HRQOL compared to that of the general population, and higher scores indicated a greater HRQOL. Sixty-six (80.49%) patients had a score lower than 50 in this study.

### 2. The influence of palliative care on the choice of place of death and rescue method at the end of life

Among the 82 patients included in this study, only 1 actively sought palliative care before admission. In addition, 9 patients received palliative care consultation after admission to the resuscitation room because they agreed with all rescue measures at admission, whereas the doctors believed these rescue measures were meaningless and would lead to additional pain and injury to the patients. The last chosen place of death was divided into two categories: the resuscitation room without company and other medical places accompanied by family. The last selected rescue methods were divided into three classes: no treatment, do not resuscitate (DNR) and agreement with all rescue measures, including cardiopulmonary resuscitation. For these patients, palliative care doctors further helped them and their families understand the condition of the disease, facilitate communication between them, and find ways to help them navigate the situation.

Patients who received palliative care before admission chose to be discharged home with pain relievers and do not resuscitate orders at admission. Among the 9 patients who received palliative care after being admitted to the resuscitation room, 4/9 (44.44%) were more likely to select a medical place of death accompanied by family at the end of life after receiving palliative care rather than the first choice of staying in the resuscitation room without any family before receiving palliative care. After receiving palliative care, 7 of the 9 patients who agreed with all the rescue measures initially opted not to be resuscitated at the end of life. The details are shown in Table 2.

**Table 2:** The difference of 9 patients who received palliative care consultation after being admitted to the resuscitation room before and after receiving consultation

	The last chosen place of death			The last selected rescue method		
	Resuscitation room	Medical care places with accompanying families	P	Agree with all rescue measures	DNR	P
Before	9	0	0.023	9	0	0.001
After	5	4		2	7	

Note: DNR: Do not resuscitation

### 3. Comparison of patients who ultimately chose different treatment schemes

As shown in Tables 3 and 10 (12.20%) patients chose to discontinue all treatment, 46 (56.10%) patients chose DNR, and 26 (31.71%) patients chose to undergo all rescue measures. There were no differences in age, sex, education level, religious beliefs or type of end-stage disease among the three groups (*P* > 0.05). Patients who agreed with all the rescue measures were more likely to be actively treated for major end-stage disease than were those who discontinued treatment in the treatment group (*P* = 0.005) or the DNR group (*P* = 0.012). Patients’ consciousness in the resuscitation room, awareness of the disease and duration of terminal disease were not significantly different among the groups (*P* > 0.05). Additionally, the presence or absence of a will and a strong desire for treatment were not significantly different (*P* > 0.05). In the treatment discontinuation group, the percentage (20%) of patients who had no children was the highest among the three groups, and 60% of the patients had 2 children. In the DNR group and all rescue measures group, most patients had 1 child (54.35% and 65.38%, respectively). HRQOL, which was assessed by the MOS SF-36 score, did not significantly differ among the three groups (*P* > 0.05).

**Table 3:** Comparison of patients who finally choose different treatment schemes

	Give up all treatment (*n* = 10)	DNR (*n* = 46)	Agree with all rescue measures (*n* = 26)	P
**Age**	64.40 ± 19.93	65.24 ± 14.45	62.62 ± 12.12	> 0.05
**Sex (Male/Female)**	7/3	26/20	13/13	0.554
**Education level of the patients**				0.569
High school degree or below, n (%)	7	32	15	
College degree or above, n (%)	3	14	11	
**Religious beliefs of the patients**				0.307
Member of Communist Party of China, n (%)	2	10	8	
Buddhism, n (%)	0	0	2	
Christian, n (%)	0	4	1	
None, n (%)	8	34	15	
**Type of end-stage disease**				0.569
Malignant tumor, n (%)	8	36	20	
Organ failure, n (%)	0	3	0	
No ability to take care of themselves, n (%)	2	7	6	
**The major end stage disease had previously been actively treated**				0.019
Yes, n (%)	6	33	25	
No, n (%)	4	13	1	
**Consciousness of the patient**				0.697
Aware, n (%)	7	26	16	
Somnolence, n (%)	1	3	1	
Confusion, n (%)	0	8	2	
Coma, n (%)	2	9	7	
**Whether the patient was aware of his or her disease**				0.456
Absolutely yes, n (%)	5	25	15	
Partially yes, n (%)	3	8	2	
Absolutely no, n (%)	1	2	4	
Unknowing, n (%)	1	11	5	
**Whether a will had been made**				0.897
Yes, n (%)	2	9	4	
No, n (%)	8	37	22	
**Whether the patient had a strong desire for treatment**				0.721
Yes, n (%)	5	22	15	
No, n (%)	5	24	11	
**Time of diagnosing terminal disease**				0.835
< 1 month, n (%)	2	4	4	
1 month to 1 year, n (%)	3	17	8	
> 1 year, n (%)	5	25	14	
**Number of children**				0.025
0	2	4	1	
1	1	25	17	
2	6	8	6	
More than 2	1	9	2	
**MOS SF-36 score**				0.484
< 50	7	39	20	
≥ 50	3	7	6	

Note: DNR: Do not resuscitation

### 4. The influence of patients’ knowledge of end-stage disease on the final choice of death place and rescue method

A total of 45 (54.89%) patients were completely aware of their disease, 13 (15.85%) patients were partially aware of their disease, 7 (8.54%) patients knew nothing about their disease, and the awareness of the disease was unclear in 17 (20.73%) patients. No differences were found in the last chosen place of death or in the last selected rescue method among the 4 groups (*P* = 0.314 and 0.456, respectively) or between patients who were aware of the disease (including all and partially informed patients, *n* = 58) and patients who were not aware of the disease (*n* = 7) (*P* = 0.131 and 0.297, respectively). The details are shown in Table 4.

**Table 4:** The influence of patients’ knowledge of the end-stage disease on the final choice of death place and rescue method

	All informed (*n* = 45)	Partially informed (*n* = 13)	Know nothing about the end-stage disease (*n* = 7)	Weather the patient know about the disease is unknown (*n* = 17)	P
**The last chosen place of death**					0.314
Resuscitation room	20	4	5	6	
Medical care places with accompanying families	25	9	2	11	
**The last selected rescue method**					0.456
Give up all treatment	5	3	1	1	
DNR	25	8	2	11	
Agree with all rescue measures	15	2	4	5	

Note: DNR: Do not resuscitation

## Discussion

Our study showed that some patients (3.91%) with end-stage disease were admitted to the resuscitation room of the ED even though the frequency was not high. However, the frequency admittance to the resuscitation room may be greater in the whole country because PUMCH is the diagnosis and treatment center for complex, severe and rare diseases, and the proportion of patients with end-stage disease is relatively low. These data were consistent with previous data [11, 12]. Resuscitation rooms can cause fear, unease and loneliness in these patients rather than effective treatment. A previous study showed that ED-based end-of-life services did not alter the dying process [13]. Palliative care in the ED is crucial for effectively communicating with patients to determine their goals and provide medical care in line with their wishes [14]. Palliative care in the ED could help doctors provide essentially clear, empathetic communication and treatment of end-of-life symptoms [14]. A survey showed that the preferences for receiving palliative care and dying at home were greater than those for receiving actual care and highlighted the service gaps for better end-of-life care [15].

Only 1 (1.22%) patient in this study actively sought palliative care before admission. Patients’ and families’ knowledge about palliative care was lacking. Education about palliative care for patients and their families may help to solve this problem [16]. Doctors and social workers from our palliative care department help patients and their families have a sufficient understanding of the condition of the disease, facilitate communication between them, help them understand each other’s true thoughts, and find ways to help them navigate the situation. Even though receiving palliative care was at the discretion of the attending physician in this study, 44.44% (4/9) and 77.78% (7/9) of patients opted for a medical place of death accompanied by family and DNR, respectively, at the end of life after receiving palliative care. These findings showed that palliative care could help patients and families handle these situations in a more thoughtful manner.

To investigate the influence factors of the choice of treatment schemes at the end of life, characteristics of the patients choosing different treatment were prepared. The actively treatment of the underlying disease was correlated with positive treatment. And the number of children seemed to have some effect on the choice. However, the patients’ knowledge of the disease showed no influence on the choice. In this study, there were 8.54% (7/82) patients know nothing about the end-stage disease even though they were dying. Whether they had a strong desire for learning about the disease was unknown. Also, in this study, HRQOL showed no statistical difference among the groups choosing different rescue methods. Whether knowing about the disease and HRQOL had impact on the last decision of patients on the rescue method deserves more study.

To investigate the factors influencing the choice of treatment scheme at the end of life, the characteristics of the patients who chose different treatments were assessed. Active treatment of the underlying disease was correlated with positive treatment. The number of children seemed to have some effect on the participants’ choices. However, patients’ knowledge of the disease had no influence on their treatment choice. In this study, 8.54% (7/82) of patients knew nothing about the disease even though they were dying. Whether they had a strong desire to learn about the disease was unknown. Additionally, in this study, HRQOL did not significantly differ among the groups receiving different rescue methods. However, the knowledge about the disease and HRQOL has an impact on the final decision of patients regarding the rescue method deserves additional study.

This study has several limitations. First, this was a single-center study, and national data could not be obtained. Second, the palliative care received by most of the patients in this study was at the discretion of the attending physician, and there was bias in terms of this judgment. Third, because of the critical conditions of the patients, many of the surveys were completed by family members, which may have affected the accuracy of the results of the assessment of patients’ subjective feelings. Finally, all patients in this study who asked to receive palliative care were included; however, the true proportion of patients willing to receive palliative care could not be confirmed.

## Conclusions

For patients with end-stage disease admitted to the resuscitation room of the ED, knowledge of palliative care was lacking. Palliative care could help end-stage patients avoid the damage caused by pointless and invasive resuscitation and die peacefully.

### Electronic supplementary material

Below is the link to the electronic supplementary material.

Supplementary Material 1

## Data Availability

The data that support the findings of this study are available from the corresponding author.

## References

[1] Feng J, Yang Y, Zheng X, Zhao C, Li H, Ji P (2022). Impact of COVID-19 on emergency patients in the resuscitation room: a cross-sectional study. J Clin Lab Anal.

[2] Samaha A, Fawaz M, Eid A, Gebbawi M, Yahfoufi N (2019). Data on the relationship between internet addiction and stress among Lebanese medical students in Lebanon. Data Brief.

[3] WHO. WHO definition of palliativecare. 2012 [Available from: http://www.who.int/cancer/palliative/definition/en/.

[4] Hughes MT, Smith TJ (2014). The growth of palliative care in the United States. Annu Rev Public Health.

[5] Yilmaz S, Grudzen CR, Durham DD, McNaughton C, Marcelin I, Abar B (2022). Palliative Care needs and clinical outcomes of patients with Advanced Cancer in the Emergency Department. J Palliat Med.

[6] Hsu HS, Wu TH, Lin CY, Lin CC, Chen TP, Lin WY (2021). Enhanced home palliative care could reduce emergency department visits due to non-organic dyspnea among cancer patients: a retrospective cohort study. BMC Palliat Care.

[7] Wang CL, Liu Y, Gao YL, Li QS, Liu YC, Chai YF (2023). Factors affecting do-not-attempt-resuscitation (DNAR) decisions among adult patients in the emergency department of a general tertiary teaching hospital in China: a retrospective observational study. BMJ Open.

[8] McEwan A, Silverberg JZ (2016). Palliative Care in the Emergency Department. Emerg Med Clin North Am.

[9] Ware JE (2000). Jr. SF-36 health survey update. Spine (Phila Pa 1976).

[10] Jang H, Lee K, Kim S, Kim S (2022). Unmet needs in palliative care for patients with common non-cancer diseases: a cross-sectional study. BMC Palliat Care.

[11] Smith AK, McCarthy E, Weber E, Cenzer IS, Boscardin J, Fisher J (2012). Half of older americans seen in emergency department in last month of life; most admitted to hospital, and many die there. Health Aff (Millwood).

[12] Barbera L, Taylor C, Dudgeon D (2010). Why do patients with cancer visit the emergency department near the end of life?. CMAJ.

[13] Chan YC, Yang MLC, Ho HF (2021). Characteristics and outcomes of patients referred to an Emergency Department-based End-of-Life Care Service in Hong Kong: a retrospective cohort study. Am J Hosp Palliat Care.

[14] Long DA, Koyfman A, Long B (2021). Oncologic emergencies: Palliative Care in the Emergency Department setting. J Emerg Med.

[15] Chung RY, Wong EL, Kiang N, Chau PY, Lau JYC, Wong SY (2017). Knowledge, attitudes, and preferences of advance decisions, end-of-Life Care, and Place of Care and Death in Hong Kong. A Population-Based Telephone Survey of 1067 adults. J Am Med Dir Assoc.

[16] Grudzen CR, Richardson LD, Hopper SS, Ortiz JM, Whang C, Morrison RS (2012). Does palliative care have a future in the emergency department? Discussions with attending emergency physicians. J Pain Symptom Manage.

