# Supplementary material for: A questionnaire survey on the implementation of palliative care in the emergency department in China
Source: BMC Palliat Care. 2024 Mar 9;23:68. doi: 10.1186/s12904-024-01407-5 (PMC10924310; doi:10.1186/s12904-024-01407-5)
Supplement: Supplementary file 1 — Supplementary Material 1 [file 12904_2024_1407_MOESM1_ESM.docx]

Hospice questionnaire

This questionnaire asks questions about your and your family's understanding of the disease and treatment options, without exposing your personal information. Do you agree to participate in this questionnaire?

1. Your patient ID ?
2. Your gender?
3. Your age?
4. Your education level：A Primary school B Secondary school C University graduate D university or above
5. Your party or religion：A Communist B Independent C Buddhist D Islamic E Other
6. The patient's end-stage disease was：A tumor B organ failure C old age D no self-care ability long-term need for care E birth defects F others
7. Major end-stage diseases have been actively treated before
8. Type of active treatment：A Surgery B radiotherapy C chemotherapy D drugs E others
9. Abandonment period：A less than six months B six months to one year C one year to three years D more than three years E never gives up
10. Whether the patient is conscious：A is clear that B is lethargic, C is lethargic, and D is unconscious
11. Whether the patient is aware of their disease：A knows everything, B knows everything, C knows nothing, D knows nothing
12. Have any of the following symptoms：A pain B nausea C dyspnea D constipation E Diarrhea F bloating G edema H sleep disorders I dysphagia J fatigue K poor appetite L bleeding M pressure ulcer
13. Life status before this illness A is completely unable to take care of himself B is semi-independent C is completely independent D takes care of others
14. The time of terminal illness: A is less than one month B is from one month to one year C is more than one year
15. Whether a will has been made
16. Whether the patient has a strong desire for treatment
17. Marital status：A Married B divorced C widowed D single
18. Have OR have no children
19. Offspring composition：
20. Whether the child has reached adulthood
21. Live alone or not
22. Who to live with：A son B daughter C grandson D spouse E babysitter F Brothers or sisters G Nursing homes and other care facilities
23. Survival as determined by consultation with a specialist：A less than one month B one to three months C three months to six months D six months to one year E is unclear
24. Access to reimbursement of medical expenses：
25. Provide cost support：A parents B brothers C sisters D daughter or son E spouse F others G self
26. Provide the financial status of the fee supporter：A has a fixed income and does not need to borrow, B has a fixed income and needs to borrow, and C has no fixed income and needs to borrow
27. The main decision-maker of current treatment
28. The education level of the main decision-makers
29. Whether the main decision-makers have religious beliefs
30. Whether there is disagreement among the decision-makers
31. Finally choose the place of death
32. Finally choose the way of rescue
33. Whether you need a hospice team
